# Low mannose-binding lectin (MBL) genotype is associated with future cardiovascular events in type 2 diabetic south asians. a prospective cohort study

**DOI:** 10.1186/1475-2840-10-60

**Published:** 2011-07-05

**Authors:** Machiel A Siezenga, Prataap K Chandie Shaw, Mohamed R Daha, Ton J Rabelink, Stefan P Berger

**Affiliations:** 1 Department of Nephrology, Leiden University Medical Center, Leiden, the Netherlands; 2 Department of Internal Medicine, Medical Center Haaglanden, the Hague, the Netherlands; 3 Department of Nephrology, Erasmus University Medical Center, Rotterdam, the Netherlands

**Keywords:** South Asians, type 2 diabetes, cardiovascular disease, Mannose-binding lectin (MBL)

## Abstract

**Background:**

South Asians have a high burden of type 2 diabetes and vascular complications. Vascular inflammation is considered central in the pathophysiology of atherosclerosis, and the complement system is thought to play an important role. Mannose-Binding Lectin (MBL), which activates the lectin pathway of complement activation, has been introduced as a risk marker of vascular damage. The present study explores the association of MBL levels, genotype and cardiovascular events in type 2 diabetic South Asians.

**Methods:**

We conducted a prospective observational study. A cohort consisting of 168 type 2 diabetic South Asians was followed for a median duration of 7.66 years. At baseline, MBL levels and genotype were determined. The association with future cardiovascular events was assessed by Cox proportional hazard regression.

**Results:**

During follow-up, 31 cardiovascular events occurred in 22 subjects (11 men, 11 women). The O/O genotype was significantly associated with the occurrence of cardiovascular events (hazard ratio 3.42, 95%CI 1.24-9.49, P = 0.018). However, log MBL levels were not associated with the occurrence of cardiovascular events (hazard ratio 0.93, 95% CI 0.50-1.73).

**Conclusions:**

In type 2 diabetic South Asians, the O/O MBL genotype is associated with cardiovascular events, although single serum MBL levels are not.

## Introduction

South Asian immigrants in Western societies have a high burden of diabetes and vascular complications [1]. Traditional cardiovascular risk factors only partially explain this increased risk [2]. Hence other factors must be involved.

Atherosclerosis, the pathologic substrate of macrovascular disease, is recognized to be an inflammatory process [3]. As a player in the inflammatory response, the complement system is thought to be involved in this vascular inflammation [4] Indeed, complement activation products have been demonstrated in atherosclerotic plaques [5].

The complement system can be activated via the classical, alternative or lectin pathway, which is activated when Mannose-Binding Lectin (MBL) binds to its target molecule. MBL binds carbohydrate moieties on microorganisms. However, endogenous MBL ligands, such as glycosylated immunoglobulins or cells exposed to oxidative stress, have also been identified [6]. MBL serum levels are primarily determined by 3 polymorphisms (B,C and D genotypes, commonly referred to as O-alleles) in the MBL gene (*mbl2*). Subjects with wild type MBL genotype (A/A) have the highest serum MBL levels, subjects with 1 variant allele (A/O) have intermediate levels and subjects with 2 variant alleles (O/O) have the lowest levels. In addition, polymorphisms in the promoter region influence the MBL level [7]. MBL is synthesized in the liver. Although intraindividual levels are relatively stable over time [8], a two-to threefold increase occurs during acute phase reactions [9].

It has recently been suggested that MBL is involved in the pathophysiology of cardiovascular damage in high-risk populations [10]. In a group of type 1 diabetic Caucasians, MBL levels were significantly higher in subjects with either a history of cardiovascular disease or diabetic nephropathy compared to subjects without these vascular complications [10]. In type 2 diabetic Caucasians high MBL levels were associated with increased mortality [11]. However, others found an association between low MBL levels and cardiovascular events [8,12]. Data on MBL in South Asians are lacking.

We hypothesized that MBL might be involved in the high incidence of cardiovascular complications in type 2 diabetic South Asians. The current study aims to explore the association of MBL levels and genotype with cardiovascular complications in type 2 diabetic South Asians. We demonstrate that a low MBL genotype is associated with cardiovascular events, while a single serum MBL level is not.

## Research design and methods

### Design of the follow-up study

We conducted a prospective cohort study. All studied subjects were recruited from a previously published study [13]. The original study population comprised 465 South Asians. At baseline, subjects that were not known with diabetes underwent a 75 g oral glucose tolerance test. Diabetes was diagnosed based on the ADA 2003 criteria. Out of 465 subjects, 168 subjects had type 2 diabetes at baseline (122 already known with diabetes, 46 newly diagnosed), and from these subjects follow-up data were collected. The study protocol was approved by the Institutional Medical Ethics Committee. All subjects provided informed consent.

Study-patients were followed up by letter and subsequently by phone. When subjects could not be traced by address or phone number in our database, general practitioners or participating family members were involved.

Follow-up data consisted of medical history with regard to cardiovascular events. Subjects were sent a questionnaire and were invited for a visit to our out-patient clinic. During this visit the questionnaire was reviewed by the main investigator (M.A.S.). Subjects not willing to visit the out-patient clinic were asked permission to collect medical data from their general practitioner. For subjects who had died during the follow-up period, cause of death and cardiovascular history was retrieved from the general practitioner. All (self-)reported events were verified by contacting the hospital in which the event had occurred.

### Measurements at baseline

Laboratory measurements at baseline included lipids, creatinine, fasting glucose, urinary albumin/creatinine ratio, high-sensitivity C-reactive protein (hsCRP), and plasma SC5b-9, the soluble end product of complement activation. Lipids, creatinine, glucose and urinary albumin/creatinine ratio were measured according to standard methods. High-sensitivity C-reactive protein was measured with a fully automated Cobas Integra 800, according to the manufacturers proceedings (Roche, Almere, the Netherlands). The variation coefficients (VC) were below 3%. Plasma levels of SC5b-9 were measured with an ELISA as described earlier [14].

### MBL genotyping

DNA was isolated routinely from peripheral blood leucocytes. MBL single nucleotide polymorphisms at codons 52, 54 and 57 of the *mbl2 *gene were typed by pyrosequencing. The detailed methodology has been published separately [15]. The MBL genotype of only wildtype allele carriers is designated as A/A and the presence of 1 or 2 variant alleles(s) (B, C, or D) is designated as A/O or O/O, respectively.

### Serum MBL levels

At baseline, serum MBL levels were assessed by sandwich ELISA as described previously [16]. In brief, 96-well ELISA plates (Greiner, Frickenhausen, Germany) were coated with the monoclonal antibody 3E7 (mouse IgG1 anti-MBL at 2.5 mg/ml), kindly provided by Dr. T. Fujita (Fuhushima, Japan). Serum samples were diluted 1/50 and 1/500 and incubated in the coated wells. MBL was detected with Dig-conjugated 3E7. Detection of binding of Dig-conjugated antibodies was performed using HRP-conjugated sheep anti-Dig Abs (Fab fragments, Roche, Mannheim, Germany). Enzyme activity was detected using 2,2'-azino-bis(3-ethylbenzthiazoline-6-sulfonic acid) (Sigma Chemical Co., St. Louis, MO)). The optical density was measured at 415 nm using a microplate biokinetics reader (EL312e; Biotek Instruments, Winooski, VT). A calibration line was produced using human serum from a healthy donor with a known concentration of MBL. Earlier studies indicated that this assay primarily detects wild-type MBL in serum and plasma and that there is a direct association with the MBL genotype and with MBL function [17].

### Definition of endpoint

Cardiovascular events were defined as the occurrence of either a myocardial infarction, Percutaneous Transluminal Coronary Angioplasty (PTCA), Coronary Artery Bypass Grafting (CABG), or sudden cardiac death. The latter was defined as a witnessed sudden circulatory arrest. The primary end-point was the time to the first cardiovascular event.

### Statistical analysis

Normally distributed variables are expressed as arithmetic mean ± 1 standard deviation. Skewed distributed variables are expressed as median with interquartile range.

Differences between groups were assessed with the independent samples t-test or the Mann-Whitney-U test for normally and not-normally distributed variables respectively. Comparison between multiple groups was performed with analysis of variance. Correlations were assessed by using Pearson's correlation and Spearman's correlation as appropriate. Associations with cardiovascular events were assessed by Cox proportional hazard regression..All tests were two-sided and the level of significance was set at 0.05.

All analyses were performed using SPSS Statistical Software Package (version 17.0; SPSS, Chicago, IL)

## Results

### Baseline analysis

At baseline, serum MBL levels and MBL genotype were determined in 168 diabetic subjects (122 already known with diabetes, 46 newly diagnosed). DNA was not available in 5 diabetic patients.

The median MBL level was 476 μg/L (IQR 143-1536 μg/L). Genotype distribution in South Asians was the same as the reported genotype distribution in Caucasians [7]. MBL levels differed significantly per genotype (P < 0.001): subjects with the A/A genotype had the highest MBL levels (median 1300 μg/L, IQR 535-2258) with the A/O genotype had intermediate MBL levels (median 160 μg/L, IQR 75-295) and subjects with the O/O genotype had the lowest MBL levels (median 74 μg/L, IQR 38-101).

MBL levels correlated weakly with BMI (r = -0.155, P = 0.014), HbA1c (r = 0.165, P = 0.034) and hip circumference (-0.169, P = 0.030), but not with sex, age, blood pressure, high-sensitivity C-reactive protein (hsCRP), smoking status, total cholesterol, fasting triglycerides, and plasma SC5b-9.

### Longitudinal analysis

Out of 168 type 2 diabetic subjects at baseline, 21 could not be traced and 13 subjects refused to participate and thus were excluded from analysis. Eighty-six subjects visited the out-patient clinic, 31 subjects did not visit the out-patient clinic but medical information was retrieved from the general practitioner, and 17 subjects had died (see below). The median duration of follow-up was 7.66 (IQR 7.48-8.10) years. Participants lost to follow-up did not differ in baseline characteristics from participants for whom follow-up data were available (table 1).

**Table 1:** Baseline characteristics of the type 2 diabetic South Asian study population

	Follow-up (n = 134)	Lost to follow-up (n = 34)	P-value
Age (years)	50.7 ± 11.2	48.9 ± 11.2	0.392
% male sex	46	45	0.886
Diabetes duration (years)	7.0 (0-13)	5.0 (0-11)	0.519
HbA1c (%)	7.7 ± 1.8	7.7 ± 2.0	0.976
Systolic blood pressure (mm Hg)	138 ± 24	140 ± 27	0.638
Diastolic blood pressure (mm Hg)	84 ± 11	84 ± 11	0.937
Urinary albumin/creatinine ratio (mg/mmol)	1.0 (0.4-4.6)	1.0 (0.4-5.0)	0.991
High-sensitivity C-reactive protein (mg/L)	3.5 (1.8-8.0)	4.8 (1.7-8.4)	0.851
Cockroft clearance (ml/min)	86 ± 27	93 ± 20	0.205
Total cholesterol (mmol/L)	5.1 ± 1.0	5.0 ± 0.9	0.666
Fasting triglycerides (mmol/L)	1.59 (1.16-2.32)	1.46 (1.24-2.13)	0.869
HDL-cholesterol (mmol/L)	1.23 ± 0.3	1.28 ± 0.3	0.954
Ratio total cholesterol: HDL-cholesterol	4.14 (3.45-5.05)	4.20 (3.10-5.0)	0.432
Body Mass Index	28.0 ± 4.7	28.4 ± 4.0	0.551
Waist-to hip ratio	0.97 (0.93-1.03)	0.99 (0.95-1.04)	0.223
% previous cardiovascular event	14	11	0.663
% current or previous smoker	45	39	0.528

During follow-up, 31 cardiovascular events occurred in 22 subjects (11 men, 11 women): 3 sudden cardiac deaths, 2 fatal and 5 non-fatal myocardial infarction, 13 percutaneous coronary interventions, and 8 coronary artery bypass graft procedures. Eight of these 22 subjects had already experienced a cardiovascular event at baseline.

Twelve subjects died due to non-cardiovascular causes. These patients were censored, none of them reached the primary end-point before dying.

**Figure 1:**
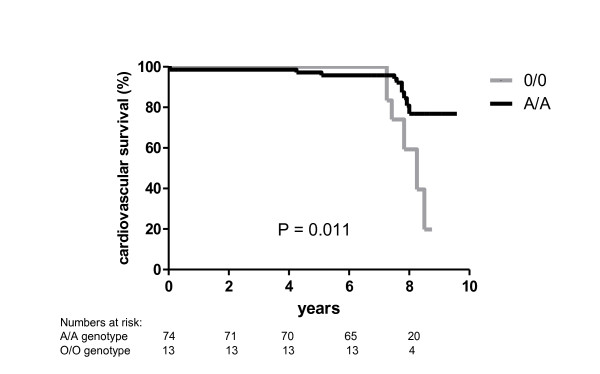
**Unadjusted Kaplan-Meier survival curves according to MBL genotype (black line = A/A (wild type), grey line = O/O genotype)**. The O/O genotype has a worse event-free survival compared to the A/A genotype (Log rank test P = 0.011).

Compared to the wild-type genotype, the O/O genotype was significantly associated with the occurrence of a cardiovascular event (hazard ratio 3.43, 95%CI 1.24-9.49, P = 0.005) (table 2 and table 3 Figure 1). Subjects with the O/O genotype did not differ in lipid parameters or blood pressure compared to subjects with the A/A or A/O genotype. The A/O genotype was not associated with cardiovascular events (HR 0.65, 95% CI 0.20-2.07). Cardiovascular events were also associated with a previous cardiovascular events (HR 4.3, 95% CI 1.2-10.3) and log urinary albumin/creatinine ratio (HR 1.58, 95% CI 1.0-2.48).

**Table 2:** Median MBL level (interquartile range in brackets) and genotype distribution in study subjects according to ethnicity and cardiovascular complications

	Median MBL level	% MBL genotype (absolute number in brackets)		
		A/A	A/O	O/O
Cross sectional				
South Asians (n = 168)	476 μg/L (143-1536)	57 (93)	35 (58)	8 (13)*
Caucasians [7]		60	36	4
				
Longitudinal				
cardiovascular event^† ^(n = 22)	390 μg/L (77-1348)	50 (11)	20 (4)	30 (7)^‡^
no cardiovascular event (112)	466 μg/L (139-1545)	56 (63)	37 (41)	7 (8)

^† ^Cardiovascular event: see text for definition

*P = 0.226 (% O/O in South Asians compared to Caucasians)

^‡ ^P = 0.004 (% O/O in subjects with cardiovascular event compared to subjects without cardiovascular event)

**Table 3:** Association with cardiovascular events of the MBL genotype and serum MBL level

	Hazard Ratio	95% CI
MBL genotype		
A/A (n = 74)	1	
A/O (n = 42)	0.65	0.20-2.07
O/O (n = 13)	3.43	1.24-9.49
Combined A/O and O/O	1.26	0.52-3.04
		
Log MBL level (per log MBL increase)		
Crude	0.93	0.50-1.73
Adjusted^a^	1.19	0.61-2.30

^a^Adjusted for urinary albumin/creatinine ratio, log high sensitivity CRP, waist-to-hip ratio, smoking status, ratio total cholesterol: HDL-cholesterol, systolic blood pressure, age, sex, HbA1c, diabetes duration

There was no statistically significant difference in baseline median MBL level between subjects experiencing a cardiovascular event during follow-up and subjects without a cardiovascular event (390 μg/L (IQR 77-1348 μg/L) versus 466 μg/L (IQR 139-1545 μg/L), P = 0.674). Log-transformed MBL levels were not associated with the occurrence of cardiovascular events (hazard ratio 0.93, 95% CI 0.50-1.73). MBL levels above the median were not associated with cardiovascular events (hazard ratio 0.94, 95% CI 0.40-2.20). Using other MBL cut-off levels also failed to show an association with cardiovascular events (data not shown).

## Discussion

From different parts of the world, South Asian ethnicity has been reported to be an independent risk factor for cardiovascular events [1,18], although survival thereafter does not seem to be worse compared to Caucasians [19,20]. We studied the effect of MBL genotype and level in a group of type 2 diabetic subjects of South Asians descent.

The main finding of the present study is that in type 2 diabetic South Asians, the O/O MBL genotype was significantly associated with the occurrence of cardiovascular events compared to wild-type.

The association between low MBL genotype and cardiovascular events has previously been reported in different populations [12,21,22]. For instance, the Strong Heart Study included American Indians [12], which - like South Asians-have a high burden of diabetes and subsequent vascular complications. A low MBL genotype was associated with a threefold increased risk for coronary heart disease.

With respect to serum MBL levels and cardiovascular events, data are more controversial. Cross sectional studies found higher MBL levels in type 1 and type 2 diabetic Caucasians with a previous cardiovascular event compared to diabetic subjects without cardiovascular disease [10,11]. In non-diabetic Caucasian males but not in females, high MBL levels were associated with future cardiovascular events [23]. In type 2 diabetic Caucasians high MBL levels were associated with increased all cause mortality, although data with respect to cardiovascular events were not reported [11]. In contrast, the prospective Reykjavik study found that in type 2 diabetic subjects low rather than high MBL levels were associated with increased incidence of myocardial infarction [8]. Recently, the Strong Heart Study provided data on MBL levels, confirming that low baseline MBL levels indeed were associated with future cardiovascular events [24]. In our study, MBL levels were not associated with future cardiovascular events.

A possible explanation for an association between low MBL levels and cardiovascular events might be a defective clearance of atherogenic particles. MBL binds N-acetylglucosamine moieties, which are expressed on several lipoproteins and oxidized LDL [25], and this may facilitate their phagocytic clearance. This hypothesis is supported by a recently published study showing that MBL deficient subjects have impaired clearance of triglyceride-rich lipoproteins [26]. Additionally, MBL deficiency might influence the susceptibility and course of infection with *Chlamydia pneumoniae*, which is associated with coronary artery disease [27]. On the other hand, MBL levels may increase in the setting of an inflammatory response [9]. Experimental studies show that in the setting of ischemia/reperfusion high MBL levels are detrimental rather than protective [28]. Oxidative stress induces a change on the cellular surface [29], which results in binding of MBL leading to enhanced complement mediated injury. However, since MBL levels were not correlated with plasma SC5-9 levels in our study, we found no evidence that high MBL levels result in increased complement activation.

Summarizing the above, whereas most cross sectional studies found an association between cardiovascular disease and high MBL levels, most prospective studies show an association between low MBL levels and cardiovascular events.

In our study, low MBL genotype was associated with cardiovascular events and MBL genotype corresponds with MBL level. One would therefore expect low MBL levels to be associated with cardiovascular events, which however was not the case in our study. MBL genotype probably is a more accurate estimate of cumulative MBL exposure than a single serum MBL level. The contribution of MBL to vascular disease might differ according to the pathophysiologic phase: early in the course low MBL levels might promote atherosclerosis, and once a vascular inflammatory response is established MBL levels might secondarily become increased and - perhaps - subsequently promote vascular inflammation. A recent experimental study demonstrated local MBL synthesis in early atherosclerotic plaques [30], supporting the hypothesis that MBL levels might become increased due to atherogenesis. Based on the assumption of time-dependency of the association between MBL and cardiovascular disease, our single baseline sample might have been too late in the pathophysiologic course to detect the effect of low MBL level on cardiovascular outcome. In addition, intra-individual variation in MBL level and variations of the MBL assay might contribute to the discordant findings between MBL genotype and MBL level. Finally, at least theoretically, the association of low MBL genotype with cardiovascular events might be based on an association with other susceptibility genes for cardiovascular events.

Noteworthy, although the O/O genotype was associated with cardiovascular events, the A/0 genotype was not, although the difference in median MBL level between the O/O and the A/O genotype was relatively small. However, it has previously been shown that although MBL concentration in A/O and O/O genotype is rather similar, functional capacity of MBL in carriers of the O/O genotype is less compared to MBL of A/O carriers. Functional MBL consists of polymers of 3 to 6 subunits. The number of structural variants influences the assembly of the MBL subunits, and lower polymerization grade results in loss of function [31].

We do want to point out that the results of our study may not apply to South Asians in general as the ancestors of the South Asians included in our study originally came from a circumscriptive area in North India called Uttar Pradesh, Uttarakhand and West-Bihar.

In conclusion, low MBL genotype is associated with cardiovascular events in type 2 diabetic South Asians, suggesting that MBL is involved in the pathogenesis of cardiovascular events. However, single serum MBL concentrations were not associated with cardiovascular events and therefore a single MBL level is not a clinically useful risk marker for cardiovascular events in type 2 diabetic South Asians.

## Abbreviations List

A/A genotype: Wild type MBL genotype; A/O genotype: 1 structural variant; HR: Hazard Ratio; MBL: Mannose-binding Lectin; O/O genotype: 2 structural variants.

## Competing interests

The authors declare that they have no competing interests.

## Authors' contributions

M.S. researched data and wrote manuscript, P.C.S researched data, M.D. reviewed manuscript, A.R.reviewed manuscript, S.B. contributed to discussion, reviewed manuscript. All authors read and approved the final manuscript.
